# Understanding the hot isostatic pressing effectiveness of laser powder bed fusion Ti-6Al-4V by *in-situ* X-ray imaging and diffraction experiments

**DOI:** 10.1038/s41598-023-45258-1

**Published:** 2023-10-27

**Authors:** Tatiana Mishurova, Sergei Evsevleev, Pierre Piault, Andrew King, Laura Henry, Giovanni Bruno

**Affiliations:** 1 Bundesanstalt für Materialforschung und–prüfung (BAM; Federal Institute for Materials Research and Testing), Unter den Eichen 87, 12205 Berlin, Germany; 2 Synchrotron SOLEIL, L’Orme des Merisiers, Saint-Aubin, 91192 Gif-sur-Yvette, France; 3 Institute of Physics and Astronomy, University of Potsdam, Karl-Liebknecht-Straße 24/25, 14476 Potsdam, Germany

**Keywords:** Metals and alloys, Imaging techniques

## Abstract

In the present study, in-situ observation of Hot Isostatic Pressure (HIP) procedure of laser powder bed fusion manufactured Ti-6Al-4V parts was performed to quantitatively estimate the densification rate of the material and the influence of the defect initial size and shape on such rate. The observations were performed in-situ using the Ultrafast Tomography Paris-Edinburgh Cell and the combination of fast phase-contrast synchrotron X-ray tomography and energy dispersive diffraction. With this strategy, we could quantify how the effectiveness of HIP depends on the characteristics of a defect. Smaller defects showed a higher densification rate, while the defect shape did not have significant effect on such rate.

## Introduction

Laser powder bed fusion (PBF-LB) additive manufacturing (AM) technology, using a layer-by-layer manufacturing approach, offers possibilities to fabricate geometrically complex parts for various industrial and medical applications^1^ at speeds and complexity levels inaccessible to conventional processing. The manufacturing process is, however, prone to generating volumetric defects inside the parts^2^. During PBF-LB three typical types of defects can be induced: lack of fusion (LoF), gas entrapped pores and keyhole pores^3^. LoF is generated by the lack of overlap between laser hatches or by insufficient energy input. Keyhole pores appear when the laser power is high enough to cause evaporation of metal at the bottom of melt pool^4,5^. Gas pores originate from bubbles of inert gas entrapped in the melt during fusion of the powder bed and/or from gas entrapped inside the feedstock material^6^.

Numerous studies on the process optimization have been conducted. They aimed at reducing the amount and volume of defects resulting from PBF-LB^3,7,8^. These optimization efforts also include *in-situ* synchrotron X-ray imaging studies conceived to understand the mechanisms of defect formation and the melt pool dynamics during manufacturing^5,9^. However, even parts produced with optimum parameter sets (i.e., avoiding the LoF and keyhole regimes) may contain some gas entrapped porosity^10^. Post-processing heat treatment (HT) is a vital step in the metallic AM production chain and is routinely used in industrial environments. Solution or stress relieving HTs are usually applied to stabilize the microstructure and alleviate the high residual stress after manufacturing^11^. However, HT is not able to eliminate defects, and therefore, they remain one of the key challenges limiting the use of AM parts in critically loaded components.

Hot Isostatic Pressing (HIP) is often introduced to tackle the porosity issue in AM materials^12^. HIP post-processing is recommended to improve the fatigue resistance of PBF-LB parts^13–15^. Even though HIP cannot completely remove porosity, it significantly decreases the defect population and its average size below the critical threshold value leading to early crack initiation. This fact allows HIPped materials to reach fatigue performances similar to those of wrought alloys^14,16^. Yand et al.^17^ have shown that a HIP temperature below 970 °C and holding time up to 2h for PBF-LB Ti-6Al-4V is sufficient to achieve mechanical properties in compliance with the ASTM standards. Owing to this fact, the efforts put in the optimization of manufacturing parameters with the goal of achieving maximum density might not be necessary when a HIP treatment is applied afterwards^3,18,19^. In this regard, Moran et al.^20^ have shown that the fatigue life of HIP PBF-LB Ti-6Al-4V is dependent on the initial material state (*i.e.*, the initial amount of defects in the as-built material). Apart from the elimination of manufacturing defects, the use of HIP has been recently proposed in the context of the shelled component densification, when a core region of the part, produced by PBF-LB^21^ or Electron Beam PBF (PBF-EB)^22^, consists of partially consolidated loose powder. This approach allows a boost of the application of HIP in AM to different production routines.

HIP is usually conducted in an inert environment by applying high temperature and pressure to shrink the internal defects by the reduction of their surface area. It has been reported to be very effective for AM materials^23^, except for the case of porosity intercepting the free surface^24^. PBF-LB manufacturing is usually performed in inert gas environment. Thus, the pores are mostly filled with inert gas, in contrast to PBF-EB process (which takes place in vacuum^23^ and the only source of gas porosity is attributed to the feedstock material). The internal pressure generated by the entrapped inert gas hinders shrinkage. In the case of PBF-LB Ti-6Al-4V components, argon is typically used as inert gas during manufacturing. However, due to its relatively large diameter, argon atoms cannot diffuse into the titanium lattice during HIP^12^ and mostly remain inside the original pores after HIP. For the case of a PBF-LB IN718 material, Xu et al.^25^ proposed that HIP was effective reducing micrometer-scale porosity, although nanometric pores still remained after the HIP cycle. From the literature review above, one can see that, up to date, the mechanisms governing the shrinkage of pores in PBF material during HIP are not fully elucidated.

In order to gain further insights into the densification mechanisms occurring during HIP, for the first time a HIP treatment of a PBF-LB Ti-6Al-4V material monitored by *in-situ* synchrotron X-ray Computed Tomography (XCT) and X-ray Diffraction (XRD) were conducted. *in-situ* observations at high pressure and high temperature are uniquely possible at the PSICHE beamline of the SOLEIL synchrotron (France), using the Ultrafast Tomography Paris-Edinburgh Cell (UToPEC) and the combination of the fast phase-contrast tomography and energy-dispersive diffraction available at PSICHE^26–29^. A detailed methodology was developed to ensure that the correct pressure and temperature were maintained during the experiments. The *in-situ* observations allowed an estimation of the global dentification rate during HIP of PBF-LB Ti-Al-4V material, as well as a detailed quantitative characterization of the influence of pore size and shape on the densification process, thereby understanding the effectiveness of HIP process on different pore categories.

## Temperature and pressure calibration and control

**Figure 1:**
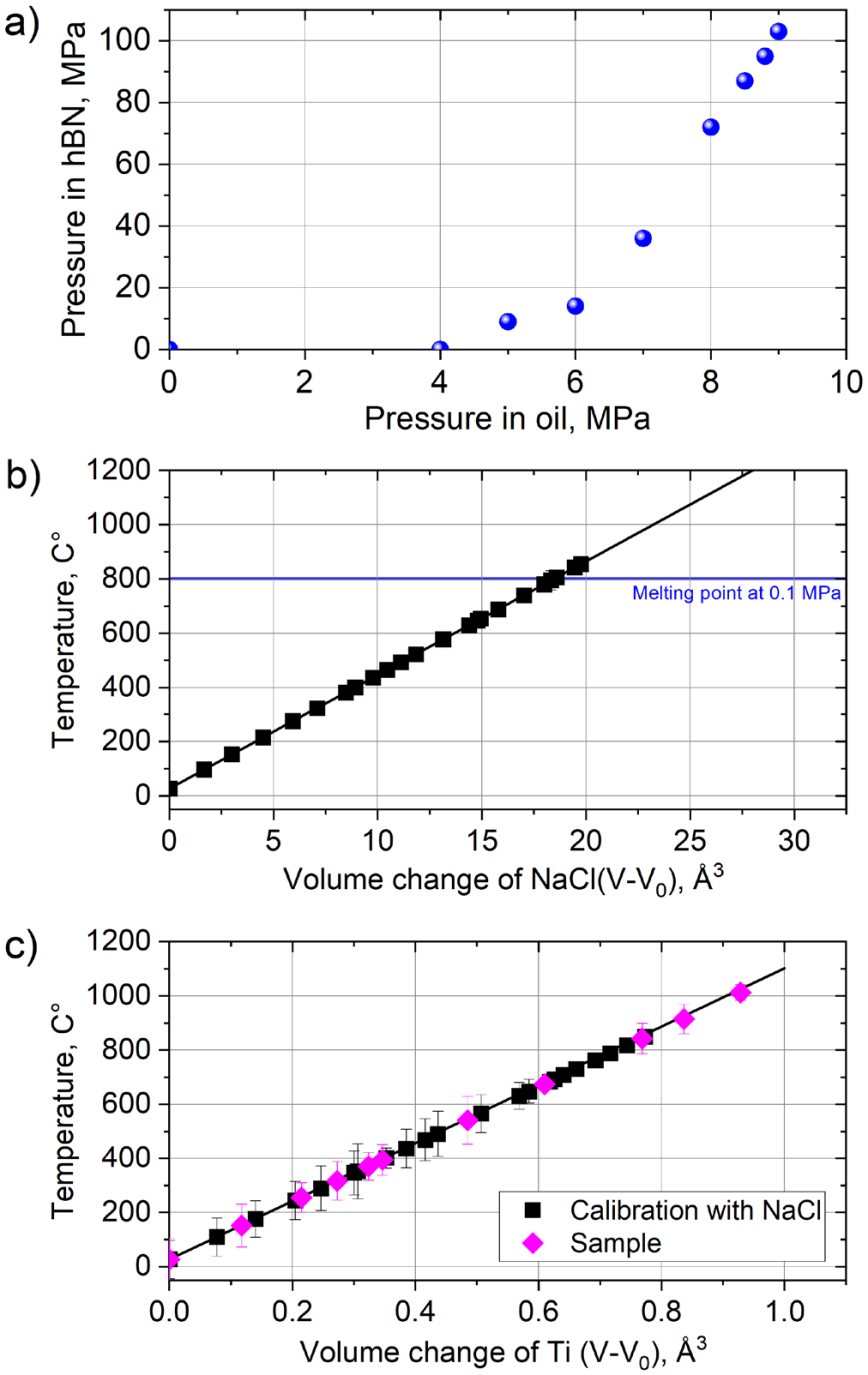
(**a**) The pressure in the hBN tube as a function of the oil pressure; and the plots showing temperature as a function of the unit cell volume change for the (**b**) NaCl during the calibration experiment, and (**c**) the investigated Ti-6Al-4 V material. The solid lines represent the linear fit of the datapoints.

For the HIP experiment, first the target pressure must be applied to the sample. The injection valve is then closed, and the high-pressure oil pump is disconnected (Fig. 1a). A pressure of around 100 MPa was targeted for the experiment. The pressure was measured using shift in the diffraction lines of hBN and the equations of state from Le Godec et al*.*^30^. After the pressure of around 100 MPa was achieved, the temperature was increased using a resistive heating via the graphite furnace. Due the relatively low applied pressure, an isobaric heating approximation was taken. In order to replicate standard HIP condition for Ti-6AL-4V, a temperature of around 920 °C had to be achieved. The temperature was calibrated using a PBF-LB Ti-6Al-4V sample and NaCl crystals. NaCl crystals were placed in a notch carved at one side of the Ti-6Al-4V samples. Since NaCl has a well-known melting point [801 °C at 1 bar (0.1 MPa)], the temperature inside the Ti-6Al-4V sample could be calibrated by monitoring the disappearance of NaCl diffraction peaks and the presence of diffuse background (Fig. 1).

The temperature ${T}_{i}$ of Ti-6Al-4V samples was calculated from the dilation of the unit cell, according to:

1 $${T}_{i}={T}_{0}+({V}_{i}-{V}_{0})/{V}_{0}\cdot {\alpha }_{V}$$

where ${T}_{0}$ is the initial temperature (RT taken as 300 K), ${V}_{i}$ is volume of unit cell at the applied temperature, ${V}_{0}$ is the initial volume of the unit cell (at RT and cold pressure), and ${\alpha }_{V}$ is the volumetric thermal expansion coefficient (CVTE).

According to the literature^31^, the linear thermal expansion coefficient ${\alpha }_{L}$ (CTE) of PBF-LB/Ti-6Al-4V is around 9.0 10^–6^ K^−1^. This value also corresponded to the CTE Ti-6Al-4V value estimated during the calibration experiment utilizing NaCl crystals (Fig. 1b). From the temperature as a function of the volume change of Ti-6Al-4V (Fig. 1c), it is observed that the Ti-6Al-4V sample follows the same trend as the NaCl calibration sample during the *in-situ* HIP experiment.

**Figure 2:**
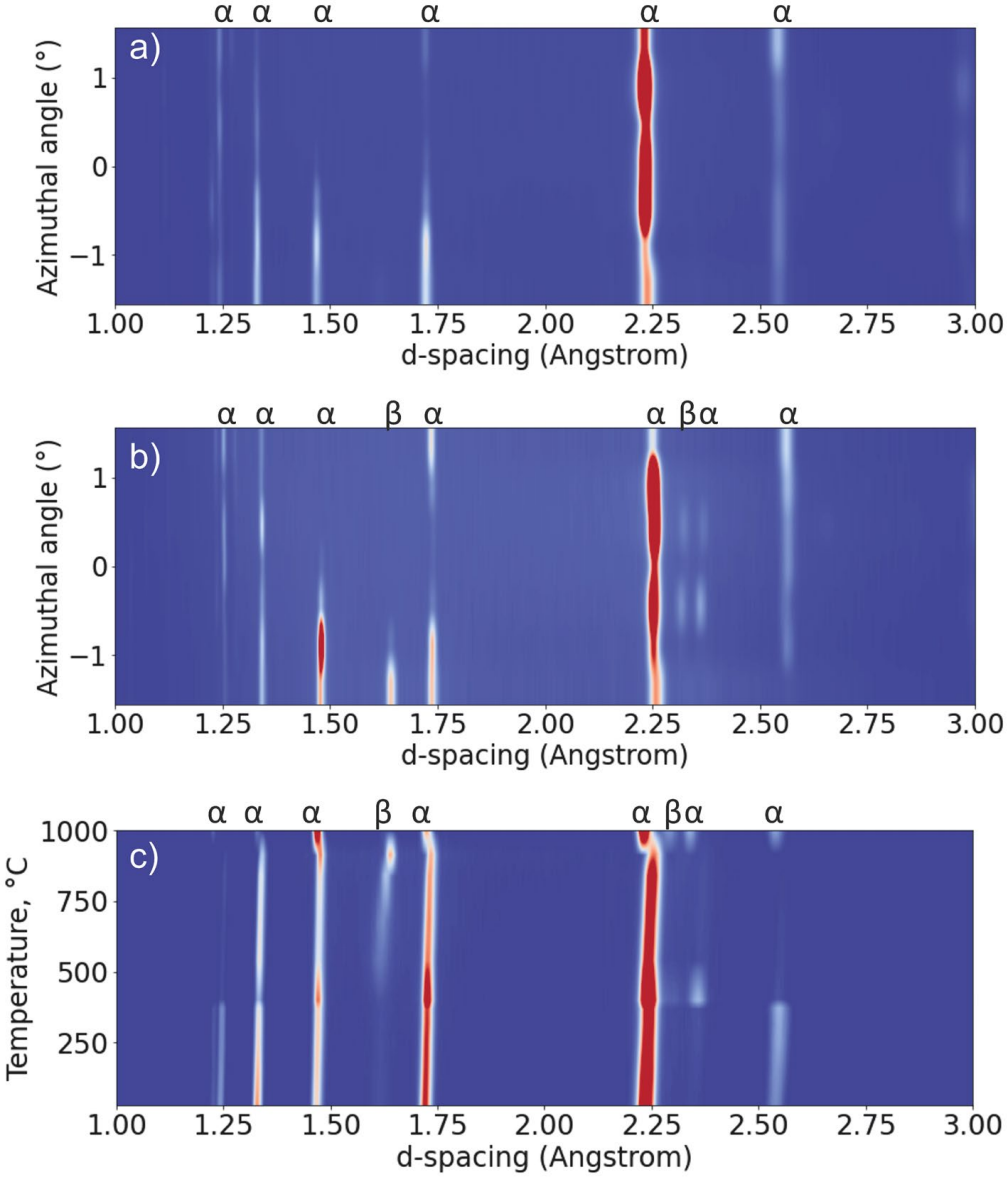
Diffraction pattern at different channels of the detector for the sample at (**a**) room temperature, (**b**) 915 °C ± 50 °C, and (**c**) the evolution of diffraction pattern during heating.

Fig. 2a,b shows the diffraction patterns for different channels of the detector (i.e., different azimuthal angles) for the sample before heating and at 915 °C, respectively, while Fig. 2c shows the evolution of the diffraction patterns during heating (for the azimuthal angle =  − 1.5°). The initial microstructure is fully α/α’, but after heating the formation of β phase can be observed.

As it is shown in^32,33^, the α phase after manufacturing presents a strong fiber texture coming from the martensitic transformation of parent β grains, having strong 〈100〉 texture along the grain growth direction^34^. Depending on the location and the orientation of the sample, the diffraction pattern may look different because of the size and orientation of α/α´ lamella packets. Thus, in the central channel (azimuthal angle = 0°) of the XRD set-up the scattering vector is oriented along the building direction of the sample. α and β phases in titanium alloys are connected thought the Burger’s orientation relationship {110}it _β_ //{0001}_α_,〈111〉_β_//〈11–20〉_α_. In the diffraction patterns (Fig. 2a,b) the 10-11_α_ peak (at d ≈ 2.25Å) is dominant (i.e. it has the maximum intensity) and no basal plane 0002_α_ peak is observed. This implies that the β-110 reflection cannot be observed. However, when changing the azimuthal angle, different grains can be observed and the β phase is detected. Thus, it can be observed that a fully α/α´ microstructure (Fig. 2a) is initially present and then transforms into α + β after the HIP experiment (Fig. 2b).

**Figure 3:**
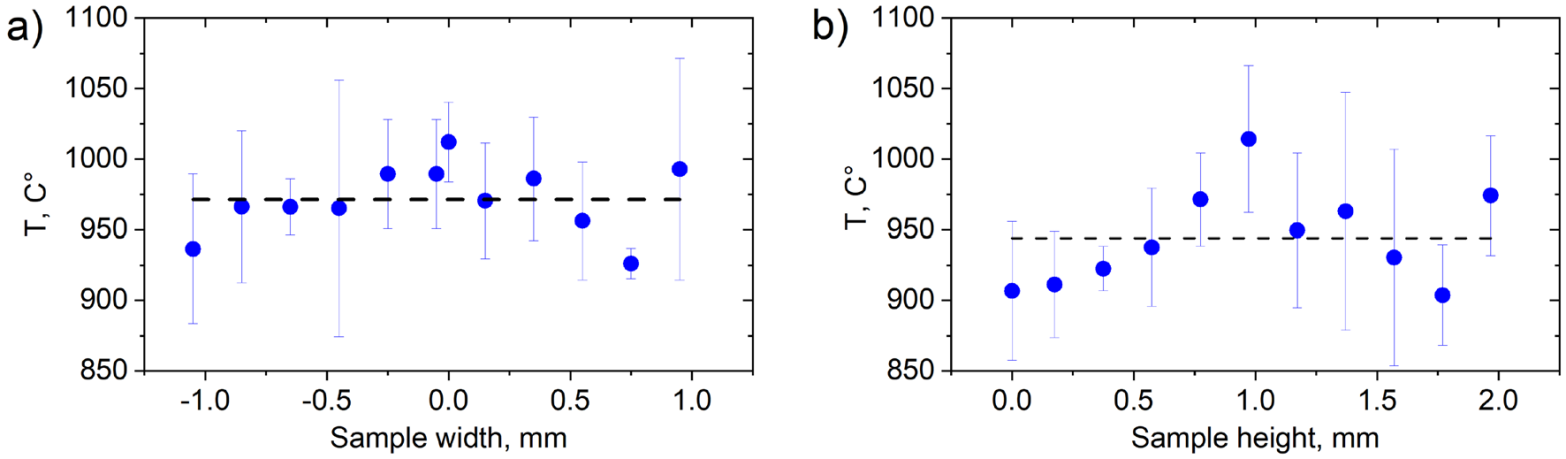
The spatial profiles of the calculated temperatures (at maximum applied heating power of 255 W) along the (**a**) width and (**b**) height of the sample.

The evolution of the microstructure with heating can be observed in Fig. 2c. According to Salsi et al.^35^, the starting temperature for martensite dissolution in Ti-6Al-4 V is around 350–400 °C, thus, an increase of β-phase content is expected above this temperature. *In-situ* diffraction observations during post-processing HT of PBF-LB Ti-6Al-4V^36^ have shown that the β-phase becomes apparent around 640 °C and the full α/α´→β transformation is completed only above 1000 °C. In the current study, the β-phase starts becoming apparent at around 500 °C (Fig. 2c). The XCT reconstructions at the initially targeted temperature of 915 °C indicated that the HIP process did not decrease the porosity amount; therefore, it was decided during the experiments to increase the temperature to 1000 °C (see Fig. 1c). To evaluate the temperature distribution inside the sample, diffraction measurements along the sample height and width were performed (Fig. 3). Results show that the temperature can vary along the sample by around 100 °C; this is further supported by finite element simulation of the temperature distribution inside a standard Paris-Edinburgh assembly^37^. Also, only partial α→β transformation was observed in the profiles in Fig. 2c, meaning that the β-transus temperature was not reached. Therefore, taking the mean value (represented be dashed line in Fig. 3) of the two measurement series we assume that a temperature around 960 °C was achieved.

## Results and discussion

**Figure 4:**
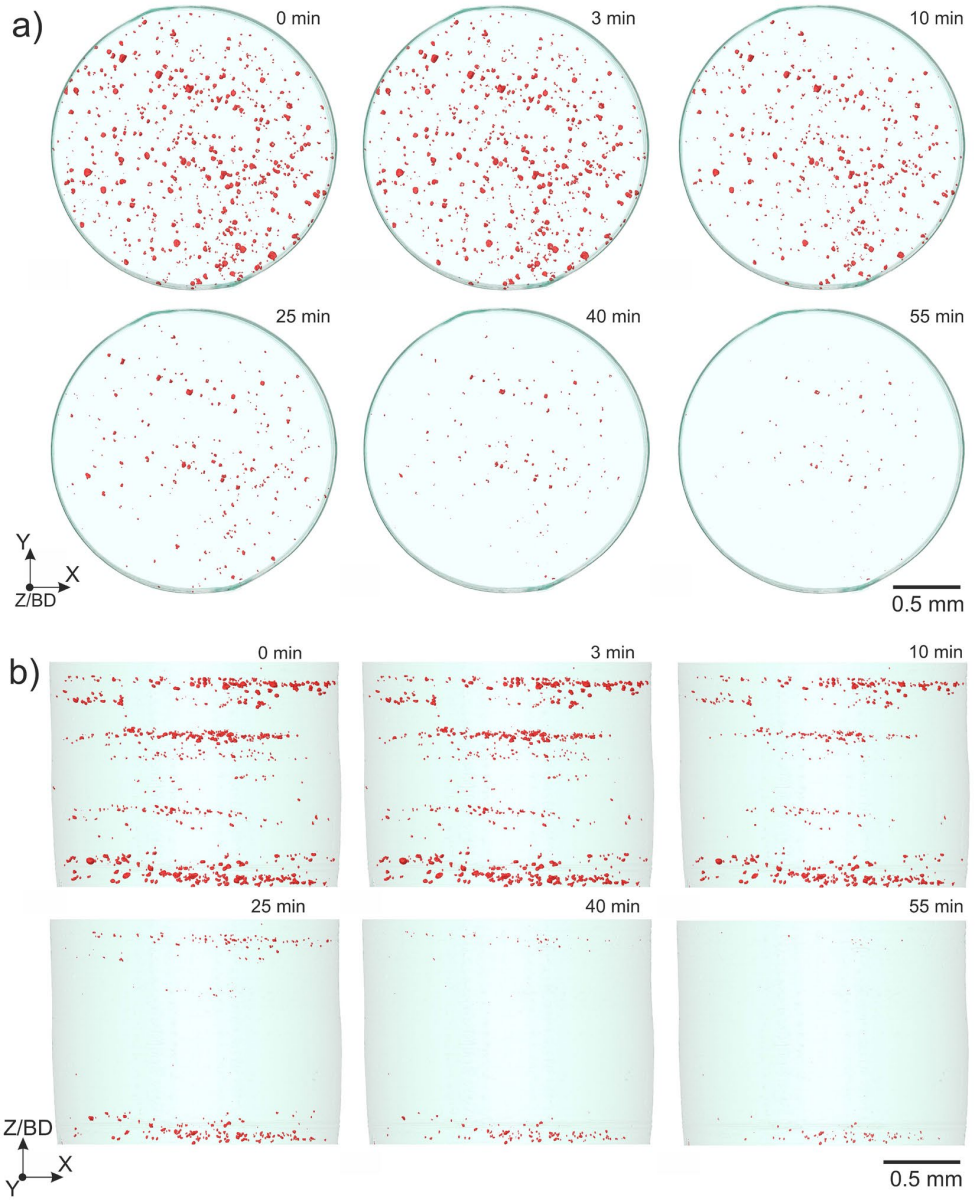
(**a**) Projection of the 3D rendering of porosity to XY plane and (**b**) to XZ plane during HIP cycle.

Fig. 4 presents the porosity 3D rendering, projected onto the XY and XZ planes (the Z axis is the build direction). Projections are shown at different steps of HIP cycle. The porosity volume fraction undergoes an immediate reduction within the first 10 min of the HIP treatment. Such decrease corresponds to both the shrinkage of larger pores and the disappearance of smaller pores. Due to the layer-by-layer build process in PBF-LB, the porosity is localised at specific heights within the sample (Fig. 4b). The densification process is more efficient at the centre of the sample. Therefore, some of the pores located closer to the bottom of the sample were not fully closed. This could be connected to the uneven distribution of the pressure from the middle of the sample to the peripheries or higher temperature in the middle of the assembly (Fig. 2b). The porosity at the periphery regions shrinks as well during the cycle but small pores are retained at the end of the cycle. Supplementary Video 1 illustrates the densification process in 3D.

**Figure 5:**
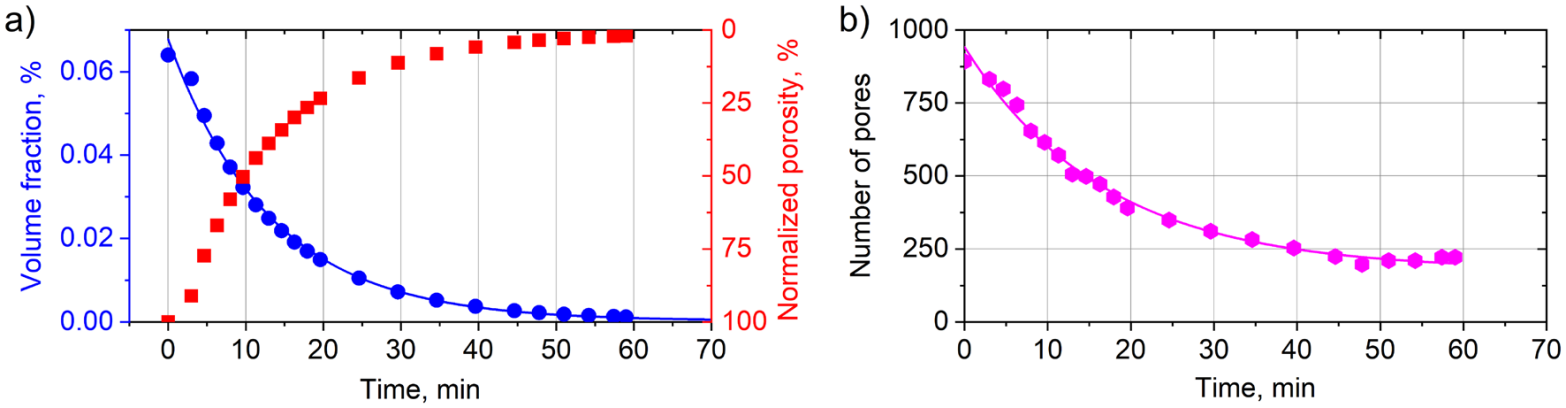
(**a**) Volume fraction and porosity normalized to the initial amount, and (**b**) the number of pores as a function of HIP time. Solid lines show the fit of exponential decay function.

Figure 5 summarizes the evolution of volume fraction and number of pores with time during the *in-situ* HIP treatment. Both the volume fraction of pores (Fig. 5a) and the number of pores (Fig. 5b) are reduced by half within about 10 min. The densification rate for volume *k*_*V*_ and number of pores *k*_*N*_ was calculated by fitting the measured points with an exponential decay function (solid line in Fig. 5a) leading to *k*_*V*_ = 0.075 ± 0.003 1/min and *k*_*N*_ = 0.059 ± 0.003 1/min. Beyond 50 to 60 min of HIP, no pronounced densification is observed, and the number of pores stays constant.

**Figure 6:**
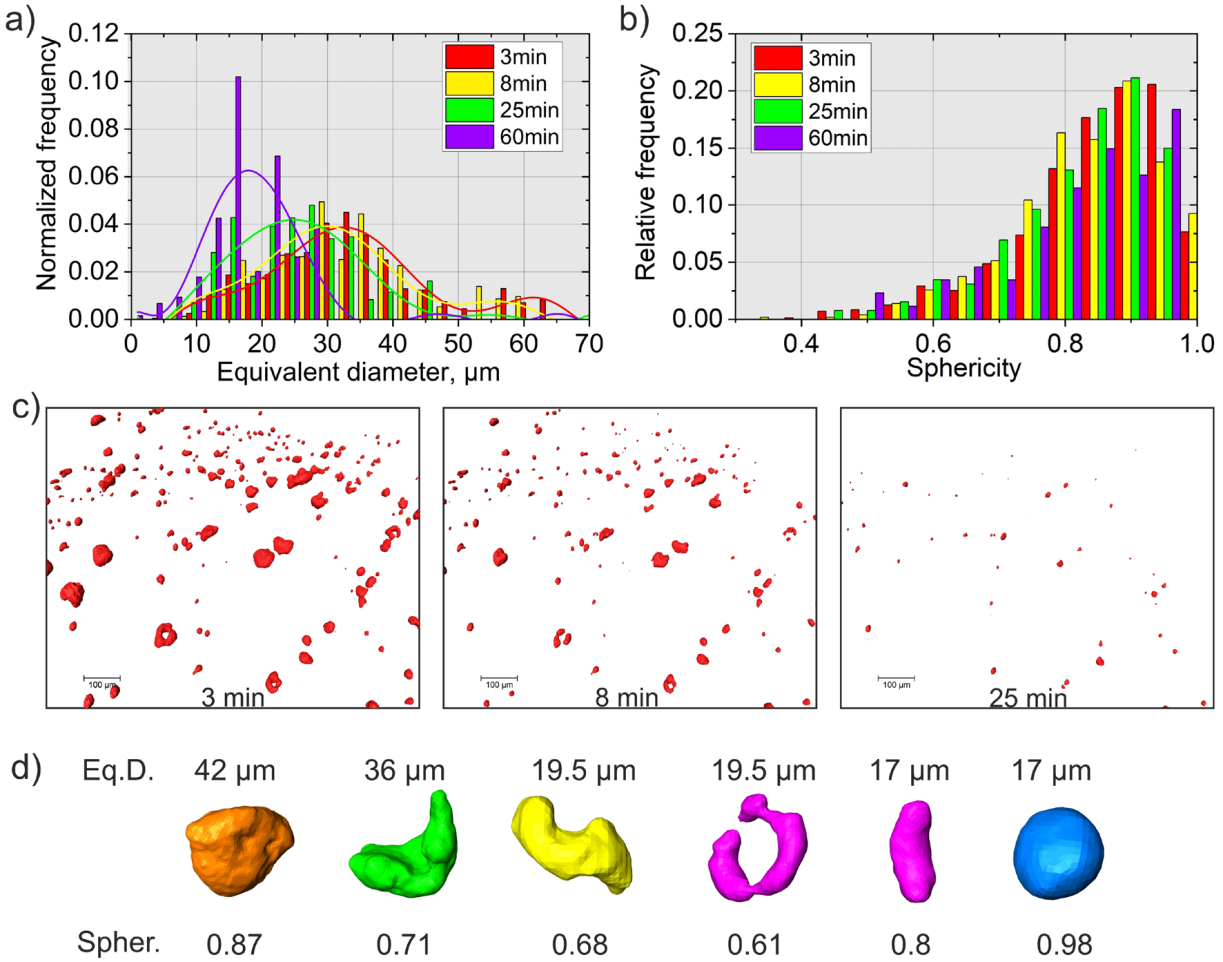
(**a**) Normalized volume-weighted frequency of porosity as a function of equivalent diameter (fitted with polynomial function), (**b**) relative frequency distribution of pore sphericity, (**c**) 3D rendering of porosity at different times of the HIP treatment, (**d**) 3D rendering of individual pores showing differences in size and shape (quantified by the sphericity).

Figure 6 shows the volume-weighted frequency distribution of the equivalent diameter (*i.e.*, diameter of a sphere having the same volume as a pore, Fig. 6a) and of the sphericity of pores (Fig. 6b) at different times during the HIP treatment. Such data represent the overall porosity, *i.e*., including all pores inside the field-of-view. Initially, two populations of pores were observed (*i.e*., two peaks can be distinguished in the distribution, Fig. 6a): one with maximum at around 35 µm and the other at 60 µm. Figure 6c shows a zoomed 3D rendering of some pores in 3D and their evolution over time. It is evident that already after 8 min of HIP some small defects disappear. After 25 min of HIP, there are no pores above 50 µm left, and the median value of the size distribution lies around 25 µm (Fig. 6a).

The sphericity ($\Psi$) is one of the parameters often used for the classification of defects (*i.e.*, to distinguish between LoF, gas pores and keyhole voids). It can be calculated for individual defects from the XCT data as

2 $$\Psi =\frac{6 \cdot {\pi }^\frac{1}{2} \cdot V}{{A}^\frac{3}{2}}$$

where *V* is the volume and *A* is the surface area of a defect.

Some researchers have classified the LoF defects as the one with sphericity less than 0.6–0.7^3,38^. As expected from the pore 3D rendering in Fig. 4, most of the pores are close to the spherical shape, having sphericity above 0.7 (Fig. 6b). However, some defects with lower sphericity can be classified as LoF defect. In the literature, LoF defects are presented as large defects (above 100 µm)^38^. Such size is, however, only to be ascribed to the relatively low resolution of laboratory XCT scans, where the detailed shape of LoF defects cannot be resolved. Indeed, Poudel et al.^6^ have reported, by using high-resolution or synchrotron XCT, as in the present study, that such LoF defects can also have relatively small sizes. Moreover, they made clear that a combination of a few geometrical parameters is required for the precise classification of porosity, *i.e.*, the size alone is not sufficient^6^. From the 3D rendering of some individual pores (Fig. 6d) it is visible that some LoF defects with an equivalent diameter less than 20 µm exhibit irregular size and relatively low sphericity (below 0.6).

**Figure 7:**
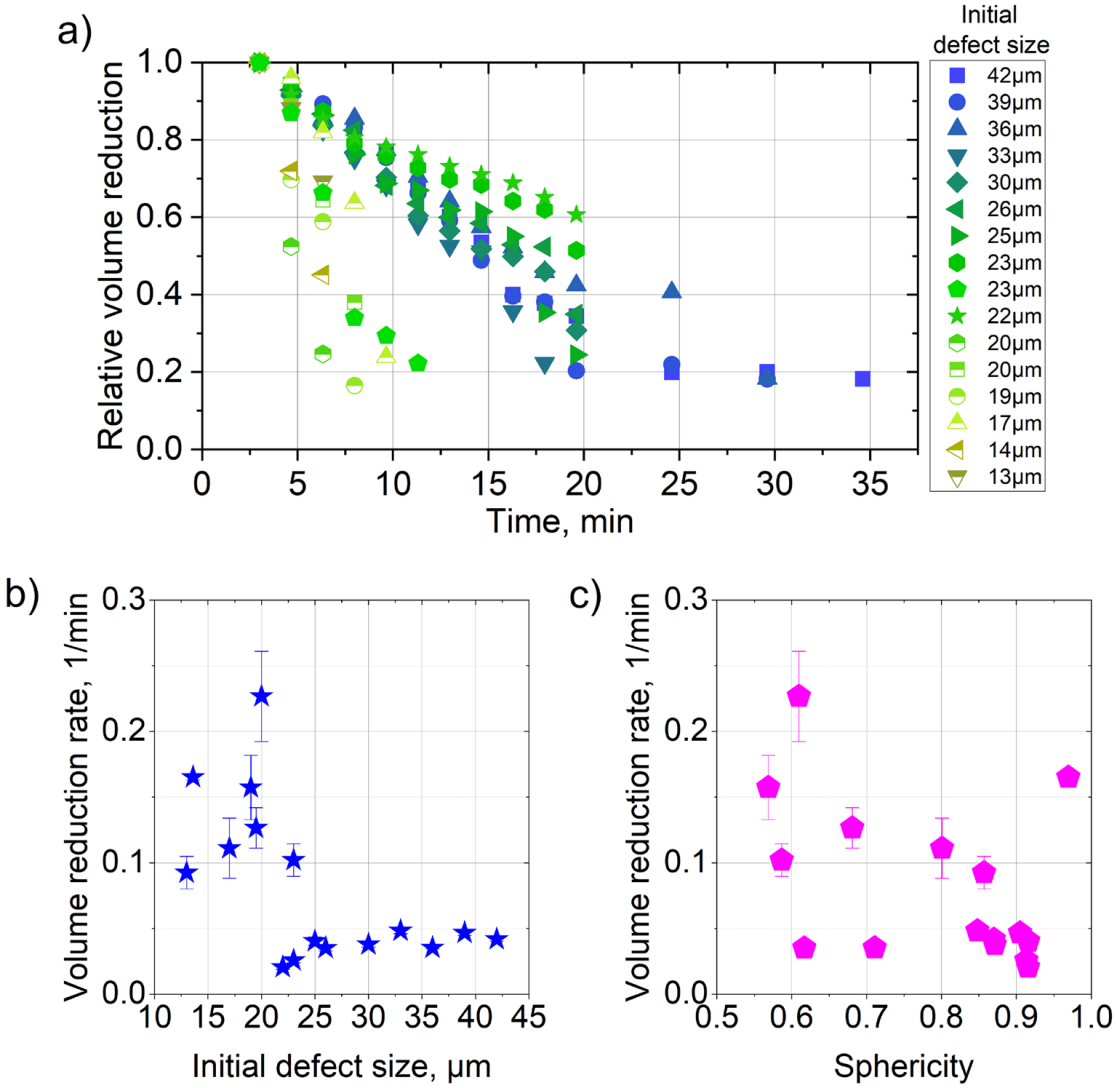
(**a**) Relative volume reduction of a defect as a fuction of time, volume reduction rate as the fucntion of (**b**) initial defect equvivalent diameter and (**c**) sphericity. Errors in (**b**) and (**c**) are calculated from the deviation from linear fit of graphs in (**a**).

Labelling of the individual defects allowed monitoring the evolution of the defect volume during the HIP treatment. This allows the estimation of the effectiveness of HIP depending on the defect characteristics. Thereby, defects with different initial equivalent diameter and sphericity were representatively chosen to estimate the effect of the (initial) defect size and shape on the densification rate (Fig. 7a). This evaluation was restricted to the defects near the center of the sample, to remove any potential effect of position within the sample on the densification rate. In Fig. 7a, the relative volume reduction represents the ratio between the defect volume during the densification process and the initial defect volume. An interruption of a graph implies that the pore disappeared (*i.e.*, its size became smaller than the resolution of the XCT with a minimum size of segmented pore of about 5 µm). First, a linear decrease of the volume with time is observed for most of the pores. After around 20 min, the size decay for the largest pores is significantly reduced. From the global volume fraction analysis, it was also confirmed that 75% of the defects vanish after 20 min of HIP (see Fig. 5).

For small times the exponential decay function (*f*(*t*) = *e*^*-kt*^) of the porosity volume reduction can be approximated with a linear function (*f*(*t*) ~ *1-kt*). Therefore, to calculate the rate of the volume reduction, the graphs were fitted using a linear function up to the point where the slope changes. The slope of the fit shows the rate of densification for different initial pore sizes (Fig. 7b,c). Interestingly, the smallest defects show the most rapid volume reduction and, thus, densify the first. While above an initial equivalent size of 20 µm the densification rate seems to be constant, we observe that the largest pores densify more rapidly than some with intermediate dimensions. This explains why the global pore *volume* decays more rapidly than the *number* (see Fig. 5).

While in general low sphericity pores tend to have a higher densification rate, no clear tendency can be inferred from the sphericity. It is concluded that the pore shape does not play a critical role in the densification rate, as much the pore size does. As proposed by Atkinson et al.^12^, the driving force to close an isolated spherical pore by HIP can be expressed in terms of pressure (P) as:

3 $${\text{P}} = {2}\upgamma /{\text{r,}}$$

where γ is the specific energy of the internal surface of the porosity and r is the radius of the sphere.

Tammas-Williams et al.^39^ have reported that the equilibrium pressure (of around 100 MPa) inside the pore of initial size of 52 µm will be achieved only when it reaches a size much below the resolution limit (of around 5 µm comparable to the present study). They concluded that possible remaining pores below 5 µm would not affect the fatigue behavior of the material, thus, making HIP an effective post-treatment for components with structural applications. The pressure inside the pore depends on the pore γ value (Eq. 3) as well as on the location of the pore. If these parameters are kept constants, according to Eq. 3, the smaller defects have larger driving force for densification. This is confirmed for most of the pores by observations the present *in-situ* XCT results (Fig. 7b). The threshold of the pore size for the change of densification rate lays in the region between 20 and 25 µm. This would imply that for the defects less than 20 µm HIP post-treatment can be very short (up to 15 min). Up to now only some simulation work^40^ could be used to give insight in the effectiveness of HIP procedure. The recent development of in-situ HIP experiment allows *experimental* quantification and validation of the simulation work. Ultimately it paves the road to tailoring the HIP procedure for different materials depending on the porosity and microstructure.

## Conclusions

*In-situ* observations using synchrotron X-ray computed tomography and energy dispersive X-ray diffraction at the PSICHE beamline, Synchrotron SOLEIL, France, allowed direct observation of the material densification during a HIP treatment (representative of those commonly applied to PBF-LB Ti-6Al-4V parts). The densification starts immediately during the HIP treatment. After 20 min, 75% of the pores can be considered as closed or has size below the resolution of the XCT reconstruction. We also observed that the smallest defects (below 20 µm) showed higher densification rate, while the defect shape did not have significant effect on such rate. We believe that present results pave the road to tailoring the HIP treatment in as a function of the initial defect size.

## Methods

### Material and sample preparation

Prismatic samples (5 × 5 × 15 mm^3^) were produced in a SLM Solutions 280HL machine using plasma atomized Ti-6Al-4V ELI grade 23 powder from AP&C, with a particle size of d_90_ < 50 μm^41^. The specimens were built directly on a baseplate preheated at 200 °C, without the need of support structures. A chess-pattern scan strategy with a minimum field size of 5 mm was applied and a fresh powder batch was used to ensure no oxygen contamination. The manufacturing parameters are specified in Table 1.

**Table 1:** Manufacturing parameters of the investigated samples.

Power	Hatch distance	Layer thickness	Velocity	Focus	Energy density	Line energy	Volume fraction porosity
W	mm	mm	mm/s	mm	J/mm^3^	J/mm	%
175	0.1	0.03	200	0	292	0.88	0.2

Since porosity is often heterogeneously distributed inside PBF-LB materials^7^, the samples were pre-characterized in order to extract smaller samples suited for synchrotron studies. As such, laboratory X-ray tomography (XCT) was performed on the original as-built samples, to subsequently extract small cylindrical samples with a diameter of 2.6 mm by means of electro discharge machining (EDM). The cylinders were again scanned using laboratory XCT, but using a higher resolution, in order to assess the initial porosity volume fraction (the values are given in Table 1). Overall, two cylindrical samples are discussed in this study: one used for temperature calibration and one for *in-situ* HIP cycle.

### In-situ synchrotron X-ray computed tomography and diffraction

#### Set-up and assembly

**Figure 8:**
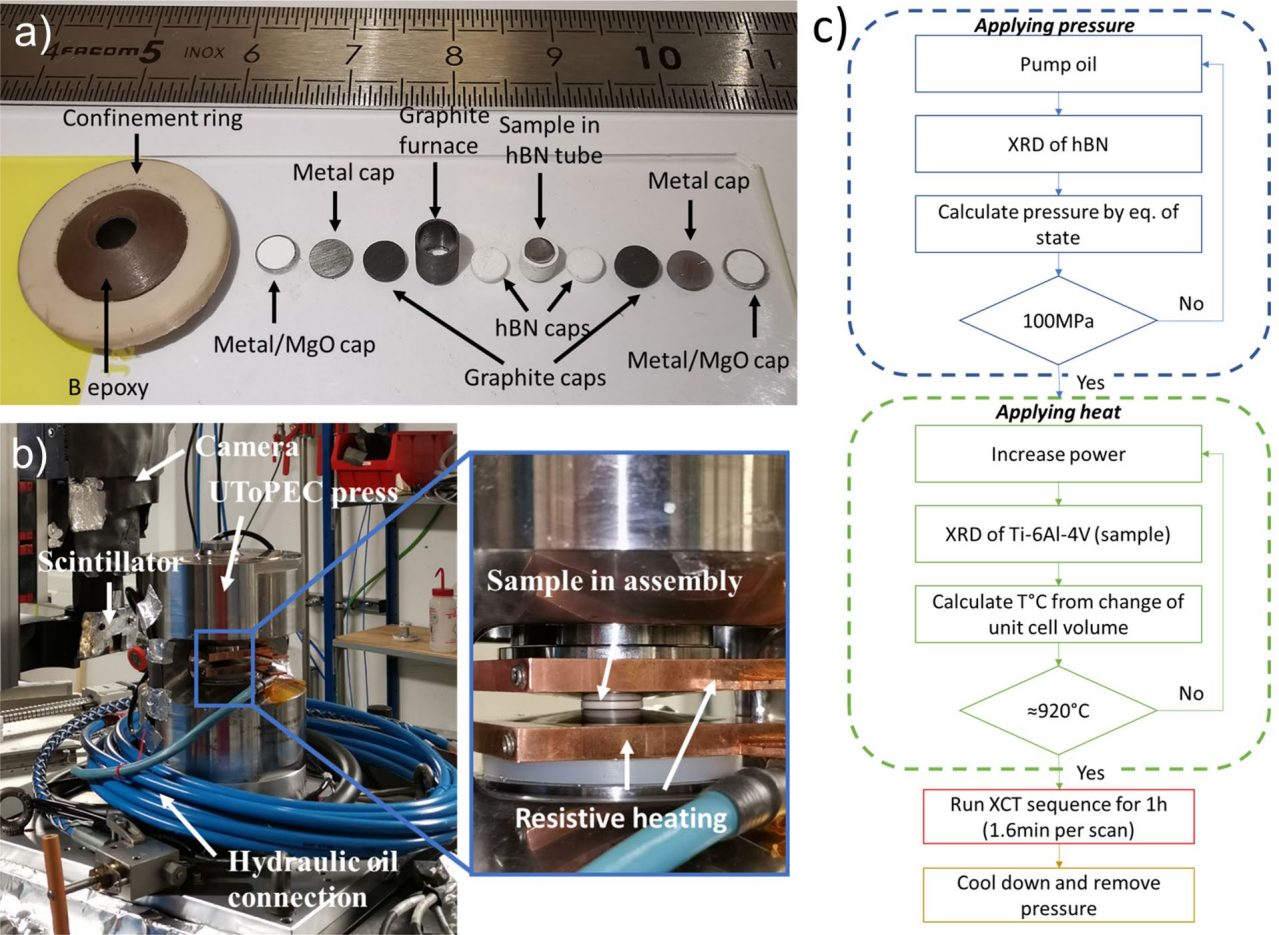
(**a**) Image showing an assembly for the sample the UToPEC press, (**b**) Image of the machine set-up used during the experiments with magnification of the High Pressure Cell (inset), (**c**) the block diagram describing the experimental procedure.

In order to perform on-the-fly (*i.e.*, the sample is continuously rotating during the scan) *in-situ* tomography at high pressure and high temperature, a Paris–Edinburgh press optimized for tomography, known as the UToPEC (Ultra-fast Tomography Paris–Edinburg Cell)^28,29^ was used at the PSICHE beamline at SOLEIL synchrotron (France), Fig. 8. For imaging, the synchrotron beam was filtered using a 0.3mm tungsten plate. The imaging system consisted of a 0.1mm thick LuAG:Ce scintillator coupled to a Hamamatsu ORCA Flash4.0 camera using a Mitutoyo 5 × microscope objective. The tungsten filter and lutetium scintillator defined an effective mean photon energy of ~ 65 keV. The detector size was 2048 × 2048 pixels, leading to the field of view of around 2.6 × 2.6 mm. The effective voxel size was 1.3 × 1.3 × 1.3 µm^3^ and the angular opening of the press was 165°. To exploit propagation-based phase contrast, the scintillator was positioned 150 mm downstream of the sample, and Paganin phase retrieval was applied to the reconstructions^42^. This set-up allows good-quality reconstructions to be obtained using the standard filtered back-projection method. To allow rotation of the press for tomography, all connections to the press (electricity for resistive heating of the sample assembly, water for cooling, electrical connections for alignment motors) pass through rotary couplings, except the hydraulic oil connection (see Fig. 8). After reaching the targeted temperature and pressure, the hydraulic connection was closed and disconnected to perform tomography. XCT scans were acquired using continuous rotation, requiring around 1.6 min per scan for all projections and reference images.

Through the conoidal shape of the boron-epoxy or pyrophyllite gasket and the use of a soft hBN pressure transmitting medium, the assembly ensure that the uniaxial stress applied by the two opposed anvils generates good hydrostatic conditions. In fact, the conical shape of the anvils allows a partial transformation of the axial stress into radial. Such shape has been optimized for high pressures and high temperatures^43^. While hydrostatic conditions cannot be reached upon cold pressurization, it was shown that upon heating at temperature of 500 K (at 4 GPa), the deviatoric stress vanishes and remains insignificant on cooling down to room temperature.

The Ti-6Al-4V samples (with the height of 2.5 mm and the diameter of around 2.4 mm) were inserted into the assembly by first placing them into the hexagonal boron nitride (hBN) capsule, then encapsulating them by a graphite furnace and covered by metallic caps for the electrical contacts to the heater (Fig. 8a). Next, they were placed in the boron epoxy gasket and placed inside the UToPEC. The cylindrical samples were initially machined slightly larger than the hBN capsule, for the samples to fit tightly to the capsule. Eventually, the samples needed to be manually polished to the required height and diameter prior the insertion into the hBN capsule.

Energy-Dispersive X-ray Diffraction (ED-XRD) was performed at each load the insitu pressure and temperature were determined using  the thermal equation of state of standard P, T markers. The Germanium Solid State Detector (Ge SSD) was placed at a nominal 2 $\theta$ angle of 8°. Prior to the experiment, the angle was calibrated using the diffraction of gold. The Ge SSD presents a multichannel analyzer which was linearly calibrated according to the fluorescence lines of several standards (Mo, Sn, Sm, Ba, Au). This provided information for azimuthal (tilt) angles in the range of about 3°. The primary slit size for the XRD experiment was 200 µm in the vertical direction and 50 µm in the horizontal direction, leading to a gauge volume length of about 1.2 mm. The diffraction data were fitted in the PDIndexer^44^, allowing the fit of the full pattern and the calculation of the lattice parameters. The diffraction measurements were performed with the gauge volume placed at the center of the sample when analyzing Ti peaks and with the press translated perpendicular to the beam when analyzing the hBN capsule. The experimental procedure is summarized in the block diagram in Fig. 8c. The procedure for heating up the sample was manual and required the calculation of the unit cell volume from diffraction patterns after each heating step. Thus, the control of the heating/cooling rate was not possible. The procedure for the calibration of the required pressure and temperature is described in detail in "Temperature and pressure calibration and control" section.

#### XCT data processing

The XCT data processing (aimed at the segmentation of porosity) required first that the reconstructed volumes be denoised. This was done using the *non-local means* filter*,* available in Fiji-Image J software^45^. The resulting volumes were cropped to maintain similar regions of interest during the whole evaluation. In fact, the field-of-view changes as a function of pressure and temperature due to vertical movements of the sample and/or closing of the gap between the anvils. Consequently, some pores could exit or enter the field-of-view during the *in-situ* HIP treatment.  The segmentation was performed using the open source Ilastik software^46^, by applying a neural network approach based on a manual segmentation of five training datasets, extracted from the data at different stages of HIP. This approach allowed avoiding the undesired segmentation of ring artefacts, which had the same grey value distribution as the targeted porosity. After segmentation, the porosity was analysed and visualized using Avizo^®^ software^47^, where the *recipe* module was applied, which allows automatic batch processing of a large number of datasets.

### Supplementary Information

Supplementary Information.

Supplementary Video 1.

## Data Availability

The datasets generated and/or analyzed during the current study are available from the corresponding author on reasonable request.
