# Supplementary material for: Understanding the hot isostatic pressing effectiveness of laser powder bed fusion Ti-6Al-4V by in-situ X-ray imaging and diffraction experiments
Source: Sci Rep. 2023 Oct 27;13:18433. doi: 10.1038/s41598-023-45258-1 (PMC10611763; doi:10.1038/s41598-023-45258-1)
Supplement: Supplementary file 1 — Supplementary Information. [file 41598_2023_45258_MOESM1_ESM.docx]

Supplementary video 1. 3D rendering of porosity evolution during HIP cycle.
